# Influence of hsCRP Parameter on the Occurrence of Metabolic Syndrome in Patients with Polycystic Ovary Syndrome

**DOI:** 10.3390/biomedicines11071953

**Published:** 2023-07-10

**Authors:** Katarzyna Lejman-Larysz, Dominika Pietrzyk, Adrianna Ćwiertnia, Mateusz Kozłowski, Sebastian Kwiatkowski, Iwona Szydłowska, Jolanta Nawrocka-Rutkowska, Jacek Brodowski, Elżbieta Sowińska-Przepiera, Aneta Cymbaluk-Płoska, Agnieszka Brodowska

**Affiliations:** 1 Department of Gynecology, Endocrinology and Gynecological Oncology, Pomeranian Medical University in Szczecin, Unii Lubelskiej 1, 71-252 Szczecin, Poland; 2 Department of Reconstructive Surgery and Gynecological Oncology, Pomeranian Medical University in Szczecin, al. Powstańców Wielkopolskich 72, 70-111 Szczecin, Poland; 3 Department of Obstetrics and Gynecology, Pomeranian Medical University in Szczecin, al. Powstaców Wielkopolskich 72, 70-111 Szczecin, Poland; 4 Primary Care Department, Pomeranian Medical University in Szczecin, Żołnierska 48, 71-210 Szczecin, Poland; 5 Department of Endocrinology, Metabolic and Internal Diseases, Pomeranian Medical University in Szczecin, Unii Lubelskiej 1, 71-252 Szczecin, Poland; 6 Pediatric, Adolescent Gynecology Clinic, Department of Gynecology, Endocrinology and Gynecological Oncology, Pomeranian Medical University in Szczecin, Unii Lubelskiej 1, 71-252 Szczecin, Poland

**Keywords:** polycystic ovary syndrome (PCOS), metabolic syndrome, high-sensitivity C-reactive protein (hsCRP), high-density lipoprotein (HDL), low-density lipoprotein (LDL), obesity, inflammatory cytokines, cardiovascular disorders

## Abstract

PCOS (polycystic ovary syndrome) is a common endocrine disorder that affects 8–13% of women of reproductive age. Increased body weight and insulin resistance may be associated with chronic inflammation, which increases the risk of cardiovascular complications. CRP (C-reactive protein) tests may be use to assess persistent inflammation. Elevated CRP levels may be associated with insulin resistance and type 2 diabetes. Determination of hsCRP, highly sensitive C-reactive protein, can be used to assess cardiovascular risk in women with PCOS. In this study, 120 women between the ages of 18 and 42 were divided into two groups: patients with polycystic ovary syndrome (*n* = 80) and regular menstruating women in whom PCOS was excluded (*n* = 40). Lipid and carbohydrate metabolism parameters and hsCRP levels were assessed, followed by receiver operating characteristic (ROC) analysis for hsCRP, where metabolic syndrome was the dependent variable. For hsCRP, the cutoff point was 1.44 (mg/dL). Sensitivity for the cutoff point was 0.913 and specificity was 0.691. The area under the curve (AUC) was 0.851 (*p* < 0.000). The closer the AUC value is to unity, the better the predictive ability of the studied variable. There was also a statistically significant correlation between hsCRP levels and the presence of metabolic syndrome.

## 1. Introduction

Polycystic ovary syndrome (PCOS) is a heterogeneous endocrine disorder that affects 8–13% of women of reproductive age [1,2]. Both genetic and environmental factors are involved in the pathogenesis of PCOS development [3]. The main feature of the syndrome is an excess of androgens produced by the ovary and/or adrenal glands [4]. Increased ovarian androgen production is caused by a modified steroidogenesis pathway and the influence of external factors such as hyperinsulinemia on the ovary [5].

Recent studies have linked chronic body inflammation, vascular endothelial damage, and oxidative stress with PCOS morbidity. Increased levels of the inflammatory cytokines Il-6 (interleukin-6), Il-18 (interleukin-18), TNF-alpha (tumor necrosis factor alpha), hsCRP (high-sensitivity C-reactive protein) and ferritin are present in patients with polycystic ovary syndrome [6]. By acting on pancreatic beta cells, TNF-alpha and interleukin 6 re-duce insulin release and directly impact the insulin receptor’s mechanism of action, which contributes to the development of receptor-type insulin resistance. They also contribute to the development of hypertension by preventing the synthesis of nitric oxide in the vascular endothelium [7]. Lower levels of factors such as adiponectin and omentin, which have anti-inflammatory properties, are also discovered [8,9].

PCOS is the most common endocrine disorder associated with obesity in women [10]. PCOS comprises a variety of metabolic abnormalities, including hyperinsulinemia, insulin resistance (IR), glucose intolerance, dyslipidemia, obesity, and non-alcoholic fatty liver disease, in addition to anovulation and reproductive disorders [11]. Inflammatory marker levels have recently been found to be higher in PCOS-afflicted women [12]. Furthermore, obesity and insulin resistance are correlated with chronic inflammation, which increases the risk of cardiovascular problems [13]. As many as 60% of patients with polycystic ovary syndrome can be diagnosed as obese, 20% of whom have a BMI > 40 kg/m^2^ (body mass index), or grade III obesity [14]. PCOS patients with obesity and those with a normal BMI had a higher waist–hip ratio (WHR) than healthy women [15]. 17 be-ta-hydroxine dehydrogenase type 5 is involved in the increased conversion of circulating androstendione to testosterone in adipose tissue in obese patients with PCOS [16]. Pro-inflammatory cytokines are also secreted at higher levels and oxidative stress parameters are also increased [17]. Acute phase proteins are a group of substances synthesized in hepatocytes under the influence of inflammatory cytokines such as interleukin-2 and interleukin-6 in the course of bacterial infections, tissue necrosis, trauma, and cancer. The most potent acute phase protein is CRP, which belongs to the pentraxin group and consists of monomers made up of 221 amino acids [18]. The CRP gene is located on chromosome 1 [19]. The infections or tissue damage that activates macrophages and causes them to release interleukin-1 and TNF-alpha begins the inflammatory cascade. Fibroblasts and endothelial cells produce interleukin-6, a key activator of the CRP protein, in response to TNF-alpha and interleukin-1 [20]. The pathophysiology of metabolic syndrome, which results in atherosclerosis and cardiovascular disease, is significantly influenced by low-grade inflammation that is activated by adipocytokines and free fatty acids.

Determination of CRP levels is therefore also used to estimate chronic inflammation that increases the risk of cardiovascular disease [21]. Furthermore, it has been shown that increased hsCRP levels are associated with arterial disease, insulin resistance and type 2 diabetes [22]. Additionally, there is a connection between carotid artery stenosis and high hsCRP levels among women [23]. 

Chronic inflammation caused by hyperinsulinemia and excessive fat accumulation in the visceral compartment underlies most of the disorders associated with PCOS. A significant meta-analysis revealed that CRP levels are nearly 100% higher in PCOS patients than in controls [24]. Serum hsCRP levels, along with the risk of cardiovascular problems, are decreased by statins, metformin, and lifestyle changes followed by weight loss [25,26]. Determination of hsCRP levels helps to estimate cardiovascular risk in women with PCOS and to guide therapeutic management in these patients [27]. The purpose of this study was to determine the effect of hsCRP levels on the incidence of metabolic syndrome in patients with polycystic ovary syndrome.

## 2. Materials and Methods

### 2.1. Participation in the Study

This is an observational study that conforms to the STROBE statement.

The study included 120 women between the ages of 18 and 42 who were hospitalized in the Department of Gynecology, Endocrinology, and Gynecologic Oncology at the Pomeranian Medical University in Szczecin, Poland between 2018 and 2021. All participants in the study were between the fifth and second days of their fertility cycle, in the early folliculotropic phase. Patients were divided into two groups:

1. Group PCOS: Patients with polycystic ovarian syndrome diagnoses (*n* = 80);
2. Group C (Control): Women menstruating regularly, without features of androgenization, in whom polycystic ovary syndrome was excluded (*n* = 40).

The study was approved by the Bioethics Committee of the Pomeranian Medical University with the number KB-0012/01/18. The Rotterdam ESHRE/ASRM (European Society of Human Reproduction and Embriology/American Society for Reproductive Medicine) criteria were used to make the diagnosis of polycystic ovary syndrome. Metabolic syndrome was diagnosed using the JIS (Joint Interim Societies) criteria and the 2009 modified IDF (International Diabetes Federation) criteria. Exclusion criteria for the study were pregnancy, breastfeeding, thyroid disease, previously diagnosed diabetes, hematological diseases, Cushing’s syndrome, congenital adrenal hyperplasia, fever, and viral or bacterial diseases. None of the patients were using hormonal contraception, hypoglycemic drugs, or vitamin D3 supplements. The definitions of PCOS and metabolic syndrome, along with the criteria for diagnosis, have been added to the methodology. A common endocrine disease called PCOS (polycystic ovarian syndrome) affects 8–13% of women of reproductive age. Chronic inflammation may be linked to increased body weight and insulin resistance, which raises the risk of cardiovascular problems. The Rotterdam consensus states that the presence of two out of three of the following criteria—oligoanovulation, hyperandrogenism, and polycystic ovaries (12 follicles measuring 2–9 mm in diameter and/or an ovarian volume > 10 mL in at least one ovary)—defines polycystic ovarian syndrome (PCOS) [28]. Metabolic syndrome is a metabolic disorder that includes cardiovascular disease risk factors such as: hyperglycemia, elevated systolic blood pressure ≥ 130 mmHg, increased waist–hip ratio, triglyceride levels above 150 mg/Dl, and HDL levels <400 mg/dL for men and <50 mgL/dL for women [29].

### 2.2. Anthropometric Measurements and Gynecological Examination

The study was conducted during hospitalization in the Department of Gynecology, Endocrinology, and Gynecologic Oncology of the Pomeranian Medical University in Szczecin, Poland. The following procedures were performed on each patient: a history; physical examination, including gynecological examination (two-handed); and speculum and ultrasound examination with a vaginal probe to evaluate the reproductive organs. The morphology of the ovaries was assessed via ultrasound, measured in three planes, and their volume was calculated using the Voluson P6 software. The severity of hirsutism was assessed using the Ferriman–Gallwey scale, as well as the presence of acne and androgenetic alopecia. Anthropometric measurements, including height, weight, and waist and hip circumference, were also obtained. Blood pressure was measured using the Korotkow method.

Each patient’s BMI (kg/m^2^) was determined (underweight 18.5, normal weight 18.5–24.99, overweight 25.0–29.99, grade I obesity 30.0–34.99, grade II obesity 35.0–39.99, and grade III obesity ≥ 40) were used to determine each patient’s BMI. On the modified Ferriman–Gallwey scale, hirsutism was deemed to be present when a patient received more than 7 points. Blood pressure was measured with a mercury pressure gauge in a sitting position, with the patient wearing a cuff sized to the width of the arm 2–3 cm above the elbow flexion. Waist circumference was measured at the end of exhalation at the narrowest point between the upper edge of the iliac crest and the lower edge of the ribcage. Hip circumference was measured at the level of the largest buttock protrusion.

### 2.3. Laboratory Studies

A 5 mL quantity of fasting patient blood was collected from the elbow vein in the morning between days 2 and 5 of the fertility cycle. The blood sample was assessed for the following parameters: hsCRP, LDL (low-density lipoprotein), HDL (high-density lipoprotein), and TG (triglycerides). In addition, the patients had their carbohydrate metabolism assessed with an oral glucose tolerance test—OGTT. After fasting, blood glucose and insulin levels were determined, and the patient was given 75 g of anhydrous glucose dissolved in 250–300 mL of water to drink within 5 min. After 120 min, during which the patient remained at rest, the patient’s serum glucose and insulin levels were again reevaluated.

The laboratory that determined those parameters accepted the following standards:

1. hsCRP: <1.0 mg/4 L—low risk of cardiovascular disease, 1.0–3.0 mg/L—intermediate risk, >3.0 mg/L—high risk;
2. LDL: <100 mg/dL, HDL: >50 mg/dL, TG: <150 mg/dL;
3. Glucose 0′: <100 mg/dL—normal, 100–125 mg/dL—abnormal fasting glucose, >125 mg/dL—diabetes;
4. Glucose 120′—<140 mg/dL normal, 140–199 mg/dL—abnormal glucose tolerance, >200mg/dL—diabetes;
5. Insulin 0′—<14 μIU/mL;
6. Insulin 120′—<60 μIU/mL.

HOMA-IR (Homeostatic Model Assessment) was calculated from the formula:

G_0_ [mg/dL] × I_0_ [umU/mL]/405

Insulin resistance is diagnosed when HOMA-IR > 2.5.

hsCRP was determined via immunoturbidimetry on a Cobas PRO module c503 instrument. 17-OHP was determined via immunoassay, ELISA on a Biotek ELX 800 reader. Insulin was determined via electrochemiluminescence (ECLIA) on a Cobas PRO module e801 instrument. HDL and LDL were determined by homogeneous colorimetric enzymatic method on a Cobas PRO module c503 apparatus. Triglycerides were measured via the enzymatic–colorimetric method, while glucose was assayed via the enzymatic method with hexokinase on a Cobas PRO module c503 apparatus.

### 2.4. Statistical Analysis

The collected data were characterized using statistical descriptors (mean, median, standard deviation, minimum and maximum values). The assumption of normality of distribution was checked using the W Shapiro–Wilk test. Intergroup comparisons of the collected data were performed using Mann–Whitney U. Correlation analysis was performed using Spearman’s R correlation coefficient. Logistic regression and ROC curve parameters were calculated to estimate the predictive values of the collected variables at the onset of metabolic syndrome.

## 3. Results

### 3.1. Characteristics of the Study Group

The study included 80 women with PCOS aged 18 to 42 years (mean age 26.3 ± 5.1 years) and 40 control women aged 18 to 46 years (mean age 27.1 ± 6.3 years). There were no significant age differences between the groups (*p* = 0.819).

Metabolic syndrome was identified in 21 patients, or 26.3% of the polycystic ovary syndrome-diagnosed women, in the cohort. Three women in the control group, or 7.5% of the group, had metabolic syndrome diagnoses. Significant differences existed between the groups (*p* = 0.015).

Women with PCOS had a mean BMI of 27.26 kg/m^2^, standard deviation 5.46. The mean value in the control group was 23.58 kg/m^2^, SD 4.41. Statistics showed that there were differences between the groups (*p* = 0.000). Compared to the control group, where it was 76.28 cm, SD 12.84, the mean waist circumference for women with PCOS was 87.08 cm, SD 17.1. *p* = 0.001 indicates that there were statistically significant differences between the groups. In the PCOS group of women, the average waist-to-hip ratio (WHR) was 0.83, SD 0.12, while in the control group, it was 0.76, SD 0.08.

The differences between the groups were statistically significant (*p* = 0.001). Women with PCOS had a mean diastolic blood pressure of 79 mmHg, with a standard deviation of 11.64. The mean value in the control group was 77 mmHg, with an SD of 8.26. Statistics showed that there were differences between the groups (*p* = 0.044). Systolic blood pressure in women with PCOS averaged 125 mmHg, with a standard deviation of 11.87. The median reading in the control group was 117 mmHg, with an SD of 12.9. Results are presented in Table 1.

### 3.2. Comparison of Carbohydrate Metabolism Parameters

The mean fasting blood glucose value in the group with PCOS was 87.13 mg/dL, SD 12.59. In the control group, the mean value was 86.66 mg/dL, SD 7.54. The mean glucose concentration value after 120 min in the OGTT test was 107.62 mg/dL, SD 34.62 in the group of patients with PCOS. In the control group, the mean value was 95.22 mg/dL, SD 23.92. The mean fasting insulin concentration value in women with PCOS was 14.76 µIU/mL, SD 16.79 and in the control group, 8.06 µIU/mL, SD 5.64. The mean insulin concentration value after 120 min in the OGTT test was 85.14 µIU/mL, SD 82.26 in the group of women with PCOS. In the control group, the mean value was 38.69 µIU/mL, SD 26.14. The mean HOMA index value in the group of women with PCOS was 3.07, SD 3.31. The mean value in the women in the control group was 1.78, SD 1.43. Results are presented in Table 2.

### 3.3. Comparison of Lipid Metabolism Parameters

In the PCOS group, the mean HDL concentration was 60.96 mg/dL with a standard deviation of 14.61. The mean result in the control group was 64.78 mg/dL, with an SD of 11.88. *p* = 0.089 indicates that there is no statistically significant difference between the groups. In the group of women with PCOS, the mean LDL content was 108.37 mg/dL, with a standard deviation of 28.45. The mean result in the control group was 96.13 mg/dL, with an SD of 30.76. Significant differences (*p* = 0.031) existed across the groups of women in the range. In the group of women with PCOS, the mean triglyceride concentration value was 96.34 mg/dL, with an SD of 60.67. The mean result in the control group was 67.75 mg/dL, with an SD of 29.3. Significant group differences occurred (*p* = 0.06). Results are presented in Table 3.

### 3.4. hsCRP

The group with PCOS had an average hsCRP level of 3.86 mg/L with a standard deviation of 6.19. The mean value in the control group was 1.0 mg/L, with an SD of 1.08. Statistics showed that there were differences between the groups (*p* = 0.000). Serum hsCRP levels of 1.0 mg/L, which are associated with a low risk of cardiovascular disease, were reported in 35% (*n* = 28) of the women in the PCOS group as opposed to 60% (*n* = 24) of the women in the control group. In the two groups, 32.5% (*n* = 26) of PCOS-afflicted women and 37.5% (*n* = 15) of the women in the control group had hsCRP levels between 1.0 and 3.0 mg/dL. Of the PCOS patients, 32.5% (*n* = 26) had an hsCRP level above 3.0 mg/dL, indicating a high risk of cardiovascular disease, compared to 2.5% (*n* = 1) of the control group.

In the group of patients with PCOS, a statistically significant positive correlation was observed between serum hsCRP levels and the occurrence of metabolic syndrome (R = 0.45; *p* = 0.000), triglyceride levels (R = 0.83; *p* = 0.001, fasting glucose (R = 0.35; *p* = 0.001), glucose after 120 min on the OGTT test (R = 0.40; *p* = 0.000), fasting insulin (R = 0.43; *p* = 0.000), insulin after 120 min on the OGTT test (R = 0.49; *p* = 0.000), HOMA index (R = 0.48; *p* = 0.000), BMI (R = 0.45; *p* = 0.000), waist circumference (R = 0.48; *p* = 0.000), WHR (R = 0.44; *p* = 0.000), systolic blood pressure (R=0.52; *p* = 0.000) and diastolic blood pressure (R = 0.37; *p* = 0.001). A statistically significant negative correlation was observed between serum hsCRP levels and levels of HDL (R = −0.49; *p* = 0.000). Results are presented in Table 4.

**Figure 1:**
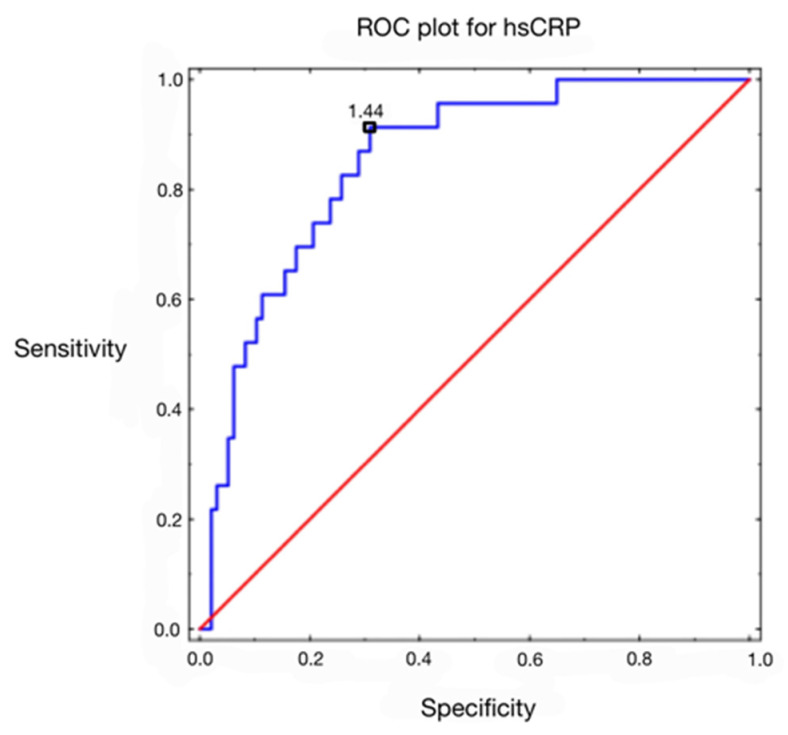
ROC curve for hsCRP (high-sensitivity C-reactive protein).

For hsCRP, a univariate logistic regression analysis was carried out with metabolic syndrome as the dependent variable. The odds ratio for hsCRP was 1.13, which indicates that the likelihood of metabolic syndrome rises by 13% for every unit increase in this parameter. Receiver operating characteristic (ROC) analysis was performed for hsCRP (Figure 1), where the dependent variable was metabolic syndrome. Cut-off points were determined for those parameters where sensitivity and specificity were most optimal. Sensitivity indicates what percentage of patients with metabolic syndrome were correctly diagnosed based on their level of hsCRP.

Specificity indicates the percentage of people correctly classified as healthy who did not have metabolic syndrome. The classification quality of the parameters was determined by the area under the curve (AUC). The closer the AUC value is to unity, the better the predictive ability of the variable under study. For hsCRP, the cutoff point was 1.44 [mg/dL]. Sensitivity for the cutoff point was 0.913 and specificity was 0.691. The area under the curve (AUC) was 0.851 (*p* < 0.000). Results are presented in Table 5, Table 6, Table 7 and Table 8.

## 4. Discussion

In this study, more than one-third of individuals with polycystic ovarian syndrome are at high risk for cardiovascular problems; patients with polycystic ovary syndrome had significantly higher mean blood hsCRP concentrations than controls. The average hsCRP level in the PCOS group was 3.86 mg/L, with a standard deviation of 6.19. The control group’s mean value was 1.0 mg/L, with a standard deviation of 1.08. Statistics revealed differences between the groups (*p* = 0.000). In the PCOS group, 35% (*n* = 28) of the women were reported as having serum hsCRP levels of 1.0 mg/L, compared to 60% (*n* = 24) of the women in the control group. This level is associated with a low risk of cardiovascular disease. For years, researchers have been trying to understand the links between inflammation and the pathogenesis of PCOS. It is not clear whether the inflammation is caused by PCOS itself or by the often coexisting obesity and accumulation of visceral fat. More than half of women with PCOS are overweight or obese with excessive fat accumulation in the visceral compartment. Adipocytes and white adipose tissue lymphocytes and macrophages are a major source of circulating proinflammatory cytokines and chemokines in these patients [30]. Cytokines such as Il-1 and TNF-alpha, by stimulating endothelial cells and fibroblasts to produce Il-6, affect CRP secretion via hepatocytes and directly in adipose tissue. In the literature, researchers note increased serum levels of hsCRP, TNFalpha, Il-18, and Il-6 in PCOS patients. Chronic inflammation promotes the development of insulin resistance, type 2 diabetes, endothelial dysfunction, and atherosclerotic lesions [31,32]. Furthermore, decreased levels of antioxidants can be observed in PCOS patients [33]. Of all inflammatory markers, hsCRP is the strongest and most significant predictor of cardiovascular indices [34]. The correlation between polycystic ovary syndrome and chronic inflammation was first pointed out by Kelly et al. in 2001 [35]. In a study with a small group of patients, they showed higher levels of CPR in women with PCOS compared to controls. Escobar-Morreale [36], in his meta-analysis, showed non-BMI-related CRP concentrations in PCOS patients twice as high as those of controls, but no statistically significant differences in Il-6 and TNF-alpha concentrations between groups. The largest meta-analysis so far in 2021 [37], where 85 scientific papers available in the literature comparing CRP concentrations in patients with PCOS (*n* = 5656) and controls (*n* = 4224) were analyzed, showed that most authors obtained consistent results–CRP values are significantly higher in patients with polycystic ovary syndrome. Nevertheless, the authors of this meta-analysis emphasize that there are insufficient data to determine the significance of elevated inflammatory parameters that could be associated with cardiovascular complications.

Serum levels of hsCRP in patients with polycystic ovary syndrome were found to be significantly higher compared to the control group (*p* = 0.000). The mean value of hsCRP in the group of women with PCOS was 3.86 mg/dL compared to the control group, where the mean was 1.0 mg/dL. Among patients with polycystic ovary syndrome, more than one-third had hsCRP levels >3.0 mg/mL, which is associated with a high risk of cardiovascular complications. In the control group, only one patient (2.5%) had an hsCRP level >3.0 mg/dl, indicating a significantly increased risk of cardiovascular complications in patients with PCOS compared to the general population. Although the majority of authors have obtained similar results [38,39,40], in the available literature there are papers in which researchers have not shown higher CRP values in patients with polycystic ovary syndrome [41,42]. Rudnicka et al. [13], in their 2019 study of 200 patients with PCOS and 105 controls in the Polish population, showed significantly higher serum CRP concentrations in women with PCOS.

Moreover, research has shown a positive correlation between CRP and BMI and insulin levels. A study by Zegher et al. analyzed the link between circulating growth-and-differentiation factor-15 (GDF15) levels and CRP in women with PCOS. By affecting receptors in the brainstem, GDF15 may help control body weight. C-reactive protein (CRP) and insulin are endogenous secretagogues of GDF15. Based on this correlation, Zegher et al. concluded that patients with PCOS may have reduced levels of GDF15 along with elevated levels of CRP and insulin, which may make it difficult to maintain a healthy body weight [43]. In this study, in a group of patients with PCOS, a statistically significant positive correlation was observed between serum hsCRP concentrations and triglyceride, HDL, glucose and insulin levels when fasting and after 120 min on the OGTT test, HOMA index, BMI, WHR, FAI coefficient, and blood pressure. Importantly, a correlation has been shown between hsCRP and the incidence of metabolic syndrome in women with PCOS. Mažibrada et al. [44], studying a group of adolescent girls with PCOS, reported increased hsCRP values in this group of patients and a correlation between hsCRP and BMI, waist circumference, LDL, HDL, and cholesterol, which is consistent with the results of their own study and is evidence of the role of adipose tissue in the development of metabolic syndrome. Verit et al. [45] showed an association of hsCRP with HDL, LDL, triglycerides, BMI, and WHR. Correlation with BMI was also shown in their work by Verit et al. and Moradi et al. [46] Samy et al. [47] and de Luca et al. [31] also pointed out the association of elevated hsCRP values with increased HOMA ratio, demonstrating the link between insulin resistance and inflammatory markers. It is estimated that 38–88% of women with PCOS are overweight or obese. In this study, patients in the control group had lower BMIs than the PCOS group. It is excessive body fat that may be the cause of subclinical inflammation and therefore elevated hsCRP levels [48]. It has been suggested that Il-6 and TNF-alpha are involved in the pathogenesis of insulin resistance and type 2 diabetes by inhibiting insulin receptor tyrosine phosphorylation and reducing insulin production in pancreatic beta cells [7,49]. Both Diamanti-Kandaraki et al. [38] and Wang et al. [26] in their studies demonstrated the effect of metformin on reducing inflammatory markers in PCOS patients. Some authors have suggested that hsCRP may be a helpful marker for the diagnosis of PCOS. Kalyan et al. [50] indicated that CRP/albumin ratio has a stronger association with polycystic ovary syndrome than FAI or HOMA.

Polycystic ovary syndrome is a disease that affects up to 13% of women of reproductive age [1]. There are about 9 million women of reproductive age in Poland, so up to a million women here may suffer from PCOS. Results observed in this study have shown that more than 30% of PCOS patients have hsCRP values > 3.0 mg, which means that nearly half a million women with polycystic ovary syndrome have twice the risk of acute coronary syndrome and three times the risk of ischemic stroke [51,52]. They also have an increased risk of insulin resistance, type 2 diabetes, and peripheral atherosclerosis.

In our study, the correlation between hsCRP and metabolic syndrome and all its components in patients with PCOS is confirmed. The magnitude of this problem poses a huge diagnostic and therapeutic challenge. The Centers for Disease Control and Prevention (CDC) and the American Heart Association (AHA) [53] in their 2004 report consider it reasonable to determine hsCRP in patients with risk factors for primary prevention of coronary artery disease and possible intensification of treatment. No recommendations that have appeared to date have included the determination of hsCRP in patients with PCOS to identify patients at high risk for cardiovascular complications. In view of the significantly higher mean hsCRP values in patients with PCOS compared with controls, and the high sensitivity of this parameter for metabolic syndrome (sensitivity = 0.913, specificity = 0.691), it seems that routine determination of hsCRP in all patients with PCOS may be warranted and needs further analysis. The other analytes considered hold promise as markers of inflammation but are constrained by their instability, lack of commercial assays applicable to the routine setting, inadequate performance, and lack of standardization. This is true even though the current discussion group has chosen to support hsCRP as the preferred inflammation marker [54]. Accordingly, means are being sought to prevent metabolic complications in patients with PCOS. One supplement being considered is crocin, whose effects on fasting glucose, insulin levels, and cardioprotective indices were demonstrated in a randomized controlled trial by Rahimi et al. [55]. In the study by Ziaei et al., 75 women with PCOS who were randomly divided into groups received 10 g/day of high-potency inulin (HPI) or oligofructose-enriched inulin (OEI) or placebo for 12 weeks. Based on the results, it was inferred that supplementation with long-chain inulin-type fructans (ITFs) may have a beneficial effect on inflammatory markers in women with PCOS [56]. The values of inflammatory markers (IL-6, TNF-α, CRP) in women with PCOS may also decrease following regular aerobic exercise [57].

## 5. Conclusions

Significantly higher mean serum hsCRP values were found in patients with polycystic ovary syndrome compared to controls; more than a third of patients with polycystic ovary syndrome are at high risk for cardiovascular complications. In addition to HOMA ratio, BMI, waist–hip ratio, waist circumference, and blood pressure in patients with polycystic ovary syndrome, there was a correlation between hsCRP levels and the incidence of metabolic syndrome. This correlation also held true for HDL, triglyceride, insulin, glucose, and glucose levels at fasting and after 120 min in an oral glucose tolerance test.

## Figures and Tables

**Table 1:** Characteristics of anthropometric parameters. The *p*-values of comparisons between the PCOS group (*n* = 80) and the control group (*n* = 40): BMI, body mass index; WHR, waist–hip ratio; WC, waist circumference; SD, standard deviation.

	Group	Mean	SD	Median	Min.	Max.	*p*
WC [cm]	PCOS	87.08	17.10	85.50	59.00	137.00	0.001
	Control	76.28	12.84	72.00	58.00	119.00	
WHR	PCOS	0.83	0.12	0.82	0.66	1.57	0.001
	Control	0.76	0.08	0.74	0.65	0.95	
BMI (kg/m^2^)	PCOS	27.26	5.46	26.05	17.70	42.00	0.000
	Control	23.58	4.41	22.65	18.80	41.20	
Systolic blood pressure (mmHg)	PCOS	125.03	11.87	125.50	90.00	145.00	0.001
	Control	117.30	12.90	119.00	80.00	142.00	
Diastolic blood pressure (mmHg)	PCOS	79.35	11.64	81.00	9.00	95.00	0.044
	Control	77.13	8.26	76.00	62.00	98.00	

**Table 2:** Characteristics of carbohydrate metabolism parameters. The *p*-values of comparisons between the PCOS group (*n* = 80), and the control group (*n* = 40): HOMA, Homeostatic Model Assessment.

	Group	Mean	SD	Median	Min.	Max.	*p*
Glucose 0′ (mg/dL)	PCOS	87.13	12.59	87.80	6.60	125.20	0.391
	Control	86.66	7.54	86.70	72.60	105.80	
Glucose 120′ (mg/dL)	PCOS	107.62	34.62	103.25	10.00	264.50	0.044
	Control	95.22	23.92	92.00	33.60	148.80	
Insulin 0′ (umU/mL)	PCOS	14.76	16.79	10.70	2.60	113.00	0.000
	Control	8.06	5.64	6.60	2.90	34.20	
Insulin 120′ (umU/mL)	PCOS	85.14	82.26	54.55	6.80	405.00	0.000
	Control	38.69	26.14	31.70	8.50	105.20	
HOMA	PCOS	3.07	3.31	2.28	0.51	23.13	0.000
	Control	1.78	1.43	1.39	0.59	8.57	

**Table 3:** Characteristics of lipid metabolism parameters. The *p*-values of comparisons between the PCOS group (*n* = 80), and the control group (*n* = 40): HDL, high-density lipoprotein; LDL, low-density lipoprotein; TG, triglycerides.

	Group	Mean	SD	Median	Min.	Max.	*p*
HDL (mg/dL)	PCOS	60.96	14.61	59.05	31.90	106.60	0.089
	Control	64.78	11.88	66.15	40.70	95.90	
LDL (mg/dL)	PCOS	108.37	28.45	105.45	52.00	170.30	0.031
	Control	96.13	30.76	92.75	34.60	175.90	
TG (mg/dL)	PCOS	96.34	60.67	74.50	29.00	397.70	0.006
	Control	67.75	29.30	62.60	32.70	164.00	

**Table 4:** Correlation analysis of hsCRP levels with the presence of metabolic syndrome, glucose, insulin, lipids, and anthropometric parameters in the PCOS group.

	PCOS		Control	
Parameter	Spearman’s R	*p*	Spearman’s R	*p*
Metabolic syndrome	0.45	0.000	0.31	0.053
Glucose 0′ (mg/L)	0.35	0.001	0.26	0.105
Glucose 120′ (mg/dL)	0.40	0.000	0.04	0.798
Insulin 0′ (umU/mL)	0.43	0.000	0.19	0.241
Insulin 120′ (umU/mL)	0.49	0.000	0.52	0.001
HOMA	0.48	0.000	0.19	0.232
HDL (mg/dL)	−0.49	0.000	−0.32	0.046
LDL (mg/dL)	0.20	0.071	0.20	0.219
TG (mg/dL)	0.38	0.001	0.02	0.902
WC (cm)	0.48	0.000	0.46	0.003
WHR	0.44	0.000	0.44	0.004
BMI (kg/m^2)^	0.45	0.000	0.38	0.016
Systolic blood pressure (mmHg)	0.52	0.000	0.25	0.113
Diastolic blood pressure (mmHg)	0.37	0.001	0.03	0.873

**Table 5:** Characteristics of hsCRP concentrations. The *p*-values of comparisons between the PCOS group (*n* = 80) and the control group (*n* = 40): hsCRP, high-sensitivity C-reactive protein.

	Group	Mean	SD	Median	Min.	Max.	*p*
hsCRP (mg/L)	PCOS	3.86	6.19	1.74	0.15	36.91	0.000
	Control	1.00	1.08	0.73	0.15	6.48	

**Table 6:** The incidence of deviations in hsCRP levels. The *p*-values of comparisons between the PCOS group (*n* = 80) and the control group (*n* = 40): hsCRP, high-sensitivity C-reactive protein.

	PCOS		Control		*p*
hsCRP	*n*	%	*n*	%	
<1.0 mg/L	28	35.0	24	60.0	0.000
1.0–3.0 mg/L	26	32.5	15	37.5	
≥3.0 mg/L	26	32.5	1	2.5	

**Table 7:** Univariate logistic regression analysis of hsCRP in PCOS patients in relation to metabolic syndrome: hsCRP, high sensitivity C Reactive Protein; OR, odds ratio; CI, confidence interval.

Parameter	Evaluation	OR	−95% CI	95% CI	*p*
hsCRP	0.122	1.13	1.03	1.24	0.011

**Table 8:** ROC curve parameters (sensitivity, specificity, area under the curve AUC) for hsCRP in relation to metabolic syndrome in patients with PCOS: hsCRP, high-sensitivity C-reactive protein.

Parameter	Sensitivity	Specificity	AUC	*p*
hsCRP	0.913	0.691	0.851	0.000

## Data Availability

The data presented in this study are available on request from the corresponding author, D.P., upon reasonable request.
